# Supplementary material for: Mutations in PCYT2 disrupt etherlipid biosynthesis and cause a complex hereditary spastic paraplegia
Source: Brain. 2019 Oct 22;142(11):3382–97. doi: 10.1093/brain/awz291 (PMC6821184; doi:10.1093/brain/awz291)
Supplement: awz291_Supplementary_Data [file awz291_supplementary_data.zip › awz291-Suppl_data/Supplementary_Data4.zip!/Supplementary information 4 - plasma lipidomics.zip!/Plasma lipidomics/Control vs Patient2/Statistical plots and data/TG[O] species/Volcano plot_alternative.pdf]

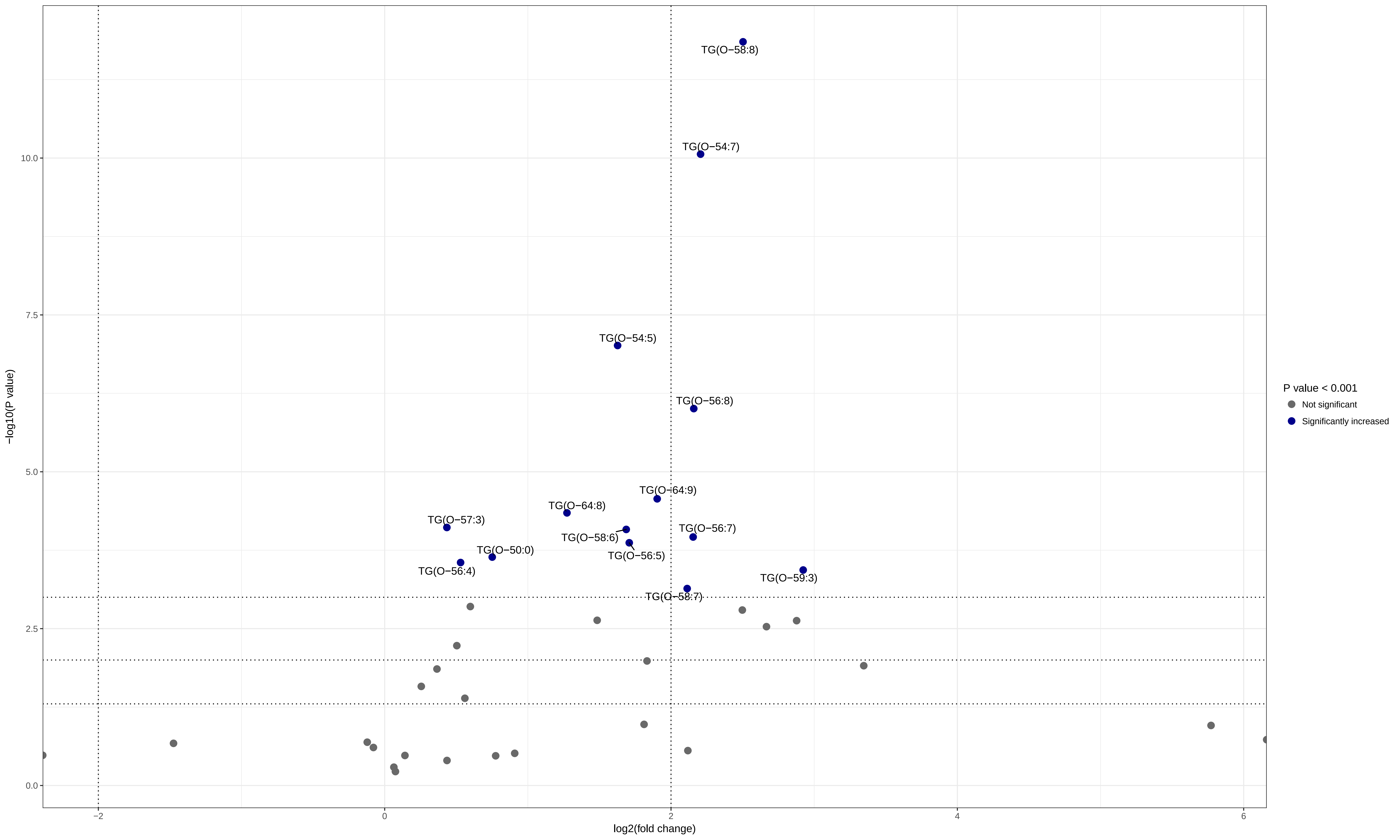
TG(O−58:8)
TG(O−54:7)
TG(O−54:5)
TG(O−56:8)
TG(O−64:9)
TG(O−64:8)
TG(O−57:3)
TG(O−56:7)
TG(O−58:6)
TG(O−50:0)
TG(O−56:5)
TG(O−56:4)
TG(O−59:3)
TG(O−58:7)
−log10(P value)
log2(fold change)
0.0
2.5
5.0
7.5
10.0
−2
0
2
4
6
P value < 0.001
Not significant
Significantly increased
